# Delivering the National Diabetes Prevention Program: Assessment of Enrollment in In-Person and Virtual Organizations

**DOI:** 10.1155/2022/2942918

**Published:** 2022-02-01

**Authors:** Michael J. Cannon, Boon Peng Ng, Kayla Lloyd, John Reynolds, Elizabeth K. Ely

**Affiliations:** ^1^Division of Diabetes Translation, Centers for Disease Control and Prevention, Atlanta, GA, USA; ^2^College of Nursing and Disability, Aging and Technology Cluster, University of Central Florida, Orlando, FL, USA; ^3^Deloitte Consulting, Atlanta, GA, USA; ^4^Cyberdata Technologies, Inc., Herndon, VA, USA

## Abstract

The aim of the US Centers for Disease Control and Prevention's (CDC) National Diabetes Prevention Program (National DPP) is to make an evidence-based lifestyle change program widely available to the more than 88 million American adults at risk for developing type 2 diabetes. The National DPP allows for program delivery using four delivery modes: in person, online, distance learning, and combination. The objective of this study was to analyze cumulative enrollment in the National DPP by delivery mode. We included all participants who enrolled in CDC-recognized organizations delivering the lifestyle change program between January 1, 2012, and December 31, 2019, and whose data were submitted to CDC's Diabetes Prevention Recognition Program. During this time, the number of participants who enrolled was 455,954. Enrollment, by delivery mode, was 166,691 for in-person; 269,004 for online; 4,786 for distance-learning; and 15,473 for combination. In-person organizations enrolled the lowest proportion of men (19.4%) and the highest proportions of non-Hispanic Black/African American (16.1%) and older (65+ years) participants (28.2%). Online organizations enrolled the highest proportions of men (27.1%), younger (18-44 years) participants (41.5%), and non-Hispanic White participants (70.3%). Distance-learning organizations enrolled the lowest proportion of Hispanic/Latino participants (9.0%). Combination organizations enrolled the highest proportions of Hispanic/Latino participants (37.3%) and participants who had obesity (84.1%). Most in-person participants enrolled in organizations classified as community-centered entities (41.4%) or medical providers (31.2%). Online and distance-learning participants were primarily enrolled (93.3% and 70.2%, respectively) in organizations classified as for-profit businesses or insurers. Participants in combination programs were enrolled almost exclusively in organizations classified as medical providers (89%). The National DPP has reached nearly half a million participants since its inception in 2012, but continued expansion is critical to stem the tide of type 2 diabetes among the many Americans at high risk.

## 1. Introduction

The Diabetes Prevention Program (DPP) trial [1] and subsequent translation studies [2–4] demonstrated that a structured, cost-effective intervention can be delivered in a real-world setting to prevent or delay type 2 diabetes among individuals at high risk [5–7]. In 2010, to accomplish widespread implementation of the 2002 DPP study results, the US Congress authorized the US Centers for Disease Control and Prevention (CDC) to create and lead the National Diabetes Prevention Program (National DPP) [8], whose aim is to make an evidence-based behavioral change intervention widely available to individuals at high risk for developing type 2 diabetes.

In 2012, CDC implemented the Diabetes Prevention Recognition Program (DPRP) as the quality assurance arm of the National DPP. The DPRP sets quality standards, collects data, and provides recognition to organizations that are delivering the National DPP lifestyle change program in accordance with the DPRP Standards and Operating Procedures (DPRP Standards) [9]. CDC-recognized organizations are required to use a CDC-approved curriculum, follow the duration and intensity requirements, and make biannual data submissions in order to maintain recognition. In order to advance to the status of being fully recognized, additional requirements involving attendance, weight loss, eligibility, and documentation must also be achieved. CDC has reported on various aspects of the National DPP's progress, including organization and participant characteristics and outcomes [10–14]. Initially, program delivery was required to be in-person, where participants are physically present with a trained lifestyle coach in a classroom or classroom-like setting. However, beginning in 2015, the DPRP began recognizing virtual delivery of the National DPP lifestyle change program via online or distance-learning delivery modes [9]. Online delivery was defined as participants logging into sessions via a computer, tablet, or smartphone, with coach interactions taking place outside of these self-paced sessions (i.e., asynchronous delivery); distance-learning delivery was defined as the coach being present in one location and participants simultaneously calling in or videoconferencing from another location (i.e., synchronous delivery). In order to increase accessibility, CDC also began recognizing program delivery consisting of a combination of any of the previously defined delivery modes [9].

The objective of this paper is to describe and analyze cumulative enrollment in the National DPP lifestyle change program, with an emphasis on assessing differences by delivery mode. This represents the most comprehensive description of enrollment to date, capturing organization and participant characteristics, including geographic location, to better understand how participants are being reached through various delivery modes. This understanding will be especially relevant as we move forward from the current context of the COVID-19 pandemic, which has disrupted previous notions of how we communicate and has created a new paradigm for what is possible in chronic disease prevention and management via virtual platform [15].

## 2. Research Design and Methods

### 2.1. Population

The National DPP lifestyle change program enrolls participants 18 years of age or older who are at high risk of developing type 2 diabetes, as defined by at least one of the following: recent blood test (fasting glucose, plasma glucose, or A1C) indicating prediabetes; a clinical diagnosis of gestational diabetes mellitus (GDM) during a previous pregnancy; or a positive screening on the American Diabetes Association/CDC Prediabetes Risk Test [9]. In addition, all participants must have had a body mass index (BMI) of ≥25 kg/m^2^ (≥23 kg/m^2^, if Asian American) at enrollment. For this analysis, we defined participants as enrollees if they met these criteria and enrolled in the program from 2012 to 2019. We chose to also include as enrollees the small number of participants (<1%) who were enrolled by organizations and met all the eligibility criteria except the BMI criterion.

### 2.2. Variables

The DPRP application for CDC recognition requires that organizations submit organization-level information such as physical address, program delivery mode (in person, online, distance learning, or combination), and organization type, which we consolidated into six groupings: (1) community-centered entities (including community YMCAs, community health centers, federally qualified health centers, senior centers, and faith-based organizations); (2) higher education/cooperative extensions (including universities/schools and business coalitions on health/cooperative extension sites); (3) government (including state/local health departments and Indian Health Service/tribal/urban Indian health systems); (4) medical providers (including hospitals, health care systems, medical groups, physician practices, and pharmacies); (5) for-profit businesses and insurers (including worksites/employee wellness programs, health plans/insurers, and for-profit private businesses); and (6) others.

Organizations seeking to obtain or maintain CDC recognition must submit biannual data that include participant sex, age, race, ethnicity, height, weight, and state of residence. They must also submit participant weight and weekly physical activity minutes collected at each session throughout the program. Because this study focused on enrollment only, we did not include an assessment of participant outcomes.

Participants could report their sex as male, female, or not reported. They provided their age in years, which we categorized into one of three age groupings: 18-44, 45-64, or 65+. For race/ethnicity, we categorized participants as Hispanic/Latino or non-Hispanic/Latino and further categorized non-Hispanic/Latinos as American Indian/Alaska Native, Asian/Asian American, Black/African American, Native Hawaiian/Other Pacific Islander, White, Multi-Racial (if they selected more than one race), and Not Reported. We calculated baseline BMI using each participant's height and the earliest session weight recorded by the organization, which allowed us to place each person into one of three BMI categories: <25 kg/m^2^ (not overweight or having obesity), 25-29 kg/m^2^ (overweight), or ≥30 kg/m^2^ (having obesity). To assess eligibility at enrollment, participants reported whether they had any of the following: a determination of prediabetes from a blood test within one year of enrollment, a clinical diagnosis of GDM during a previous pregnancy, or a positive screening on the ADA/CDC Prediabetes Risk Test.

### 2.3. Data Analysis

We conducted descriptive analyses of participants by sex, age category, race/ethnicity, baseline BMI category, and eligibility category, stratified by delivery mode. Additionally, we examined the number enrolled by delivery mode and year, percentage enrolled by organization type and delivery mode, and percentage enrolled by organization type and year. We also calculated the number of CDC-recognized organizations by delivery mode and year.

We created maps to display the geographic distribution of cumulative enrollment and recognized organizations. Participant state of residence began to be collected in 2015. For records submitted before 2015, we assigned the participant's state of residence as the state where the headquarters of the participant's organization was located. Because the only approved delivery mode before 2015 was in person, we felt that this was a reasonable assignment. For each year, we estimated the enrollment per million residents by state as the total number of enrollees in that state divided by the total number of residents of the state for that year based on US Census data [16]. We estimated the cumulative number of organizations per million residents by county as follows. First, using each organization's headquarters location zip code and unique identification code, we estimated the cumulative number of organizations per zip code, which we summed for each county to get the cumulative number of organizations per county [17]. We then divided by the total number of residents per county to get the cumulative number of organizations per million residents for each county. Using the 5-digit Federal Information Processing Standards codes (FIPS) of county and Arc Map 10.6.1, we created a county-level map to display the geographical variation. We conducted all data analyses using SAS Enterprise 7.1.

## 3. Results

Between January 2012 and December 2019, 455,954 participants enrolled in the National DPP lifestyle change program. Enrollment by delivery mode was 166,691 for in person; 269,004 for online; 4,786 for distance learning; and 15,473 for combination. The most common participant characteristics were female sex, age 45-64 years, non-Hispanic White race/ethnicity, and BMI in the obesity range; more than half of participants had a blood test in the prediabetes range or history of GDM (Table 1). Several differences in participant characteristics by delivery mode are worth noting. In-person organizations enrolled the lowest proportion of men (19.4%) and the highest proportions of non-Hispanic Black/African American (16.1%) and older (65+ years) participants (28.2%). Online organizations enrolled the highest proportions of men (27.1%), younger (18-44 years) participants (41.5%), and non-Hispanic White participants (70.3%) and the lowest proportion of participants who entered the program with a blood test in the prediabetes range (34.2%). Distance-learning organizations enrolled the lowest proportion of Hispanic/Latino participants (9.0%). Combination organizations enrolled the highest proportions of Hispanic/Latino participants (37.3%), participants who had obesity (84.1%), and participants who entered the program with a blood test in the prediabetes range (86.8%).

**Figure 1:**
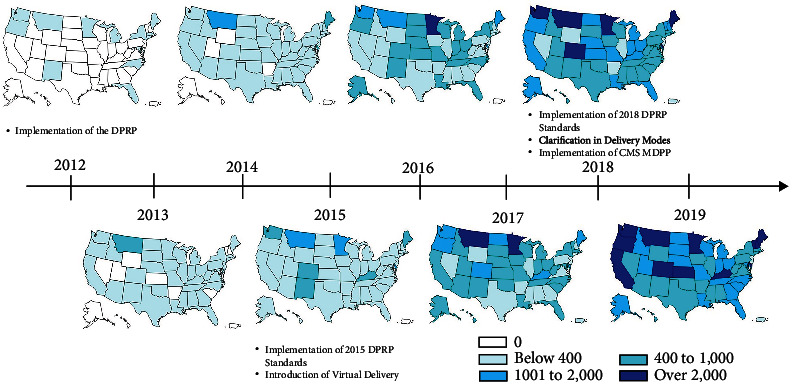
Cumulative National Diabetes Prevention Program (National DPP) enrollment from 2012 to 2019, per 1,000,000 state population. DPRP: Diabetes Prevention Recognition Program; CMS MDPP: Centers for Medicare and Medicaid Services Medicare Diabetes Prevention Program.

Figure 1 shows cumulative enrollment per 1,000,000 residents, by state, for each year since the National DPP was implemented in 2012. One key milestone for the National DPP was the introduction of virtual (online and distance learning) delivery in 2015, which resulted in expanded enrollment throughout the US. Another key milestone was the implementation of the Medicare Diabetes Prevention Program (MDPP) in 2018, which allowed for Medicare reimbursement to in-person organizations that were approved as MDPP suppliers. As of December 2019, the National DPP had reached all 50 states, along with Guam, Micronesia, Palau, Puerto Rico, and the US Virgin Islands. Cumulative enrollment has varied by state, with the highest per capita enrollment (>2,000 per 1 million) in California, Colorado, Delaware, Kansas, Kentucky, Maine, Minnesota, Montana, New Hampshire, Oregon, Vermont, and Washington.

**Figure 2:**
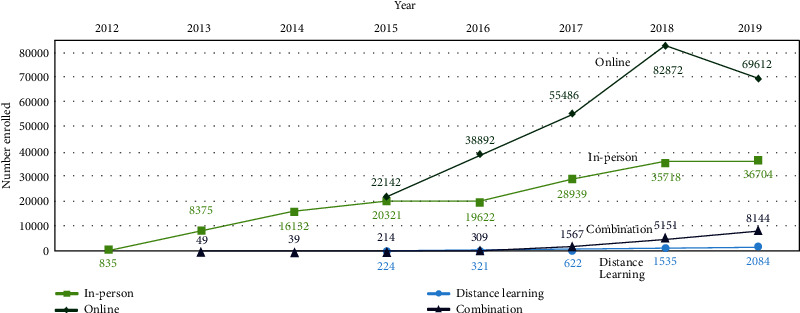
National Diabetes Prevention Program (National DPP) enrollment per year by delivery mode. For organizations that changed delivery mode from in person to distance learning or combination after 2015, we reclassified their pre-2015 data as such for consistency.

The contributions to enrollment of each delivery mode are depicted in Figure 2. In-person organizations were the first to deliver the National DPP lifestyle change program, and their yearly enrollment has gradually increased since 2012. Online organizations began delivery in 2015, creating a spike in enrollment that peaked in 2018. Distance-learning and combination delivery modes have enrolled far fewer participants but increases in distance-learning enrollment became apparent in 2018 and 2019. In 2015, three in-person organizations changed their delivery mode to combination. Participants enrolled in these organizations were retroactively classified as being in combination delivery mode for the years 2013 and 2014, explaining why the graph shows participants enrolled in combination organizations before 2015.

**Figure 3:**
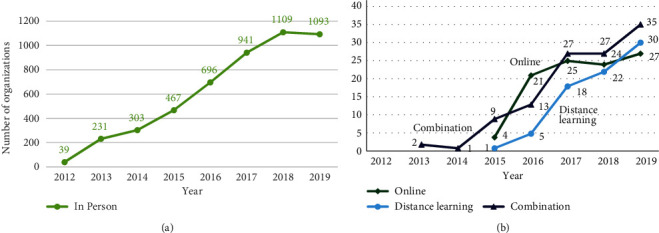
Number of recognized Diabetes Prevention Recognition Program (DPRP) organizations that enrolled participants in a given year for (a) in-person organizations; (b) online, distance-learning, and combination organizations.

National DPP enrollment has been driven by an increase in CDC-recognized organizations that deliver the program (Figure 3). The number of organizations delivering in person has increased dramatically from 39 in 2012 to more than 1,000 in 2018 and 2019 (Figure 3(a)). Similarly, the number of organizations delivering the program via other modes has increased (Figure 3(b)), although there are far fewer of these organizations.

**Figure 4:**
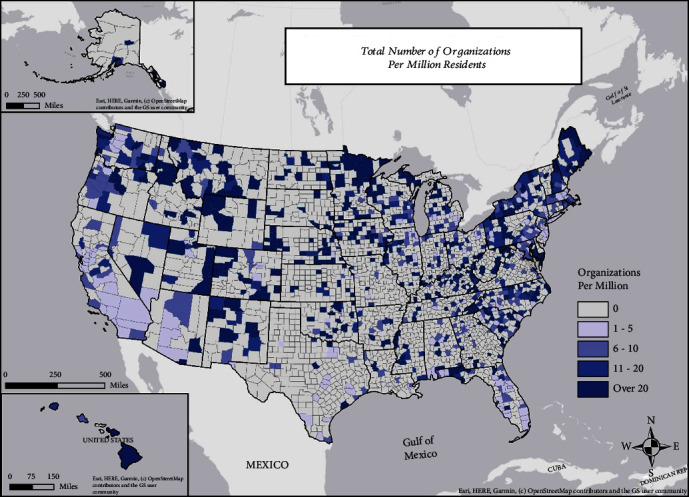
Cumulative number of Centers for Disease Control and Prevention- (CDC-) recognized organizations that enrolled participants in any year from 2012 to 2019, per 1,000,000 county population. Organizations are plotted in the map according to the county of their headquarters; it is possible that these organizations also deliver the program at other locations and to some participants who reside in different counties or states.

The National DPP lifestyle change program has been delivered by CDC-recognized organizations based in numerous counties throughout the US (Figure 4). However, the number of organizations per capita exhibits substantial geographic variability. Some counties have more than 20 organizations per million residents, while many counties have none. This absence of organizations occurs primarily in rural counties, though 52% of urban counties also show no organizations and approximately 14% have only 1-5 organizations per million residents (e.g., Maricopa County-Phoenix, Cook County-Chicago, Dallas County, and Los Angeles County).

**Figure 5:**
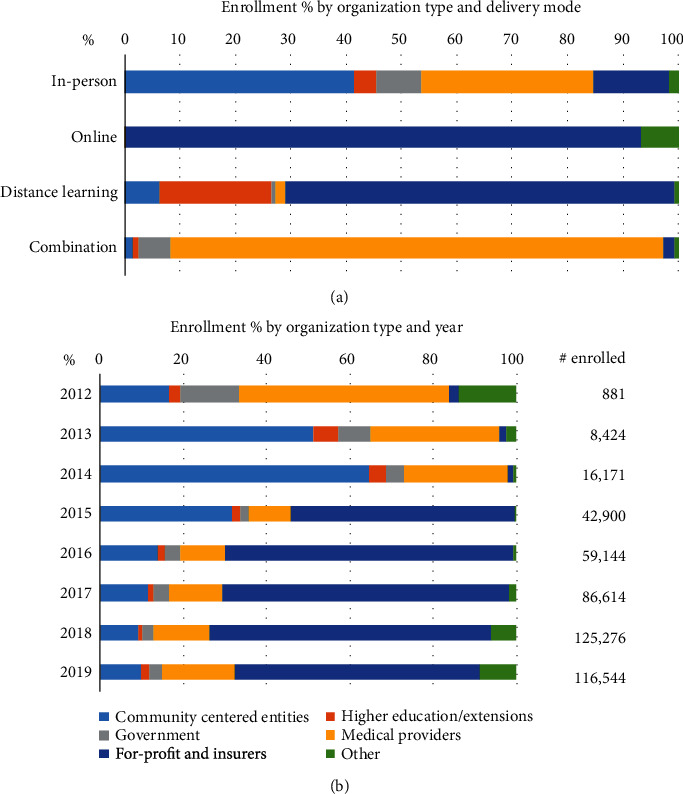
Cumulative National Diabetes Prevention Program (National DPP) enrollment percentage from 2012-2019 by (a) organization type and delivery mode; (b) organization type and year.

Different delivery modes tended to be used by different types of organizations (Figure 5(a)). Most in-person participants enrolled in organizations classified as community-centered entities (41.4%) or medical providers (31.2%). Online and distance-learning participants were overwhelmingly enrolled (93.3% and 70.2%, respectively) in organizations classified as for-profit businesses or insurers. Participants in combination programs were enrolled almost exclusively in organizations classified as medical providers (89%).

The percentage of participants enrolled in each organization type has changed over time (Figure 5(b)). During the first three years of the DPRP, when all participants were enrolled in in-person organizations, enrollment was heavily associated with community-centered entities and medical providers. When CDC started recognizing online and distance-learning organizations in 2015, there was a large increase in enrollment through for-profit businesses and insurers. Due to the expansive reach of virtual organizations, the percentages associated with for-profit businesses and insurers have remained high.

## 4. Discussion

The purpose of this study is to analyze enrollment in the National DPP lifestyle change program for each of the recognized delivery modes. The program enrolled nearly 500,000 participants from 2012 through 2019. The addition of online and distance-learning delivery modes to the DPRP in 2015 immediately increased enrollment. Although the number of CDC-recognized organizations delivering the lifestyle change program using online or distance-learning delivery modes is relatively low, their reach and ability to scale up have led to more participants enrolling through these organizations compared to in-person organizations. Although not preferred by all, virtual delivery modes allow convenience and access that appeal to many participants [18]. Furthermore, a number of research studies indicate that the lifestyle change program can be delivered effectively via virtual modes [19–23]. In future analyses, we plan to examine how key program outcomes (e.g., retention, physical activity minutes, and weight loss) vary by delivery mode in real-world settings as reflected by DPRP data.

We found some heterogeneity in the characteristics of participants enrolled through different delivery modes. Organizations using in-person delivery enrolled a higher proportion of older (65+ years) participants, whereas online and distance-learning organizations enrolled a higher proportion of younger participants (18-44 years), which may be due to their availability through workplace settings. Making virtual programs more accessible and attractive to older participants may help those who face transportation challenges or other barriers to in-person gatherings. Although some older participants may be reluctant to adopt new technologies, virtual delivery approaches have shown promise with this age group [24]. Online organizations enrolled a somewhat higher proportion of men than in-person organizations and thus may be an important avenue for increasing the relatively low proportion of men who enroll in the National DPP lifestyle change program [13]. The organizations with no in-person component (i.e., online and distance learning) enrolled lower proportions of participants who were Hispanic/Latino or non-White, suggesting that in order to reduce health disparities, these delivery modes need to be made more accessible and appealing to minority racial and ethnic groups [25]. Overall, however, we found that the racial/ethnic distributions of enrollees (Table 1) and of the US population [26] were roughly similar (13.2% vs. 17.7% for Hispanic/Latino, 64.6% vs. 61.6 for non-Hispanic White, 13.1% vs. 12.1% for non-Hispanic Black/African American, 0.9% vs. 1.0% for American Indian/Alaska Native, 3.1% vs. 5.4% for Asian/Asian American, 0.8% vs. <1.0% for Native Hawaiian/Other Pacific Islander, and 0.7% vs. 2.5% for Multiracial), although outreach could be improved for some racial/ethnic groups.

Encouragingly, the National DPP lifestyle change program has now been delivered by CDC-recognized organizations in all 50 US states, as well as in multiple U.S. territories and freely associated states. However, the differences in enrollment by state are substantial, and many counties still do not have CDC-recognized organizations. Some of these differences are mitigated by the burgeoning availability of virtual delivery; however, most online and distance-learning enrollment has been through organizations that do not have publicly available offerings; i.e., they are only available through employers or insurance plans. Therefore, program expansion to underserved rural counties and urban centers must be a future priority.

The growth in the number of CDC-recognized organizations delivering the National DPP lifestyle change program suggests that many organizations find value in the program. However, at the time of this analysis, approximately 18% of organizations ever recognized by the CDC had voluntarily discontinued their participation in the DPRP. Anecdotally, organizations report various reasons for voluntarily withdrawing from the DPRP. The most common reasons are a change in organizational priorities, frequent turnover in staffing, and lack of funding to sustain delivery of the program. Other research suggests the level of third-party reimbursement rates is an important driver of whether or not organizations offer the program [27]. In particular, in early 2018, there was a surge in new in-person organizations joining the DPRP because the Medicare benefit was being implemented. We observed that some of these organizations dropped out in 2019 when they were unable to enroll enough participants to start a cohort and submit data. To better support recognized organizations and continue to attract new organizations to the DPRP, additional research might further explore why organizations offer the program or discontinue the program.

Our data show that particular delivery modes are more likely to be used by certain types of organizations. For example, community-based organizations account for a large percentage of in-person enrollment. These organizations tend to have physical structures, such as community centers or YMCAs, that accommodate in-person delivery. Online and distance-learning organizations tend to be for-profit private businesses and typically deliver the program through employer wellness offerings or insurance benefits. One implication is that virtual programs may not be reaching individuals who are unemployed/self-employed, who do not receive employer wellness benefits, or who do not have private insurance. Reaching such individuals may require an expansion of in-person programs as well as innovative approaches to make virtual programs more affordable.

An assessment of delivery modes is especially relevant given the COVID-19 pandemic, during which the capacity and willingness to interact virtually in new ways have expanded, particularly with regard to the utilization of telehealth to consult with health care providers [28, 29]. Our findings suggest that the National DPP is well situated to capitalize on the opportunity to expand the use of virtual delivery modes and can be a leader in leveraging these modes for behavioral change for improved health.

Our study had several important limitations, the most prominent being that the DPRP collects only limited information on organizations and participants. These limitations are by design; the DPRP seeks to minimize the data collection burden to organizations. As a result, we had only a small number of participant demographic characteristics and did not have information such as participant income, employment status, or insurance status. Furthermore, until the implementation of the 2015 DPRP Standards, the DPRP did not collect information on participant state of residence. Thus, it is possible that some participants resided in states or counties that were different from where their organization was located. Although this location difference might be expected for virtual organizations, it could also have occurred for in-person organizations, not only because some participants might travel across boundaries to attend sessions but also because many organizations have multiple cohorts whose locations are not required to be reported to the DPRP. As a result, the number of organizations per million residents might have been underestimated or overestimated for some counties. In addition, we did not have any data on who was offered or referred to the program, so differences in enrollee characteristics by delivery mode could have been caused by a number of factors, including personal choice, payer source (e.g., private insurance vs. Medicaid), differences in where the delivery modes were offered (e.g., workplace vs. retirement centers), or other factors. Lastly, we did not know why most participants chose to enroll; a question about reasons for enrollment was added to the 2018 DPRP standards, but for many participants, responses were missing. A more complete picture of drivers of enrollment, therefore, will likely require research studies that collect additional information to supplement the information submitted to the DPRP.

## 5. Conclusions

The National DPP has reached nearly half a million participants since its inception in 2012, using an evidence-based approach that is proven to prevent or delay type 2 diabetes among at-risk individuals [1]. Despite this success, it has only reached a fraction of the 88 million American adults who have prediabetes [30]. Reaching more people with the National DPP will require multipronged and innovative strategies to address challenges associated with participant and health care provider awareness, access to programs, payment issues, and organizational capacity. Assessing the various strengths of the different delivery modes can help organizations choose the best one for them to help overcome some of these challenges.

## Figures and Tables

**Table 1:** Characteristics of eligible^a^ participants who have enrolled^b^ in the National Diabetes Prevention Program (National DPP) lifestyle change program, by organization delivery mode.

	Total		In-person		Online		Distance-learning		Combination	
	*N* = 455,954	%	*N* = 166,691	%	*N* = 269,004	%	*N* = 4,786	%	*N* = 15,473	%
Sex^c^										
Men	109,725	24.1	32,343	19.4	72,794	27.1	961	20.1	3,627	23.5
Women	345,641	75.9	133,964	80.6	196,021	72.9	3,824	79.9	11,832	76.5
Age group (years)										
18-44	148,179	32.5	31,543	18.9	111,682	41.5	1,464	30.6	3,490	22.6
45-64	245,222	53.8	88,107	52.9	146,224	54.4	2,846	59.5	8,045	52.0
65+	62,553	13.7	47,041	28.2	11,098	4.1	476	10.0	3,938	25.5
Race/ethnicity^d^										
Hispanic/Latino	55,338	13.2	22,446	15.6	27,412	10.7	354	9.0	5,126	37.3
Non-Hispanic/Latino	363,035	86.8	121,602	84.4	229,234	89.3	3,565	91.0	8,634	62.7
American Indian/Alaska Native	3,871	0.9	2,208	1.5	1,579	0.6	15	0.4	69	0.5
Asian/Asian American	13,087	3.1	2,653	1.8	9,642	3.8	110	2.8	682	5.0
Black/African American	54,700	13.1	23,121	16.1	28,726	11.2	624	15.9	2,229	16.2
Native Hawaiian/other Pacific Islander	3,355	0.8	1,475	1.0	1,725	0.7	92	2.3	63	0.5
White	270,330	64.6	81,885	56.8	180,537	70.3	2,558	65.3	5,350	38.9
Multiracial	2,892	0.7	1,709	1.2	1,096	0.4	39	1.0	48	0.3
Not reported	14,800	3.5	8,551	5.9	5,929	2.3	127	3.2	193	1.4
Baseline body mass index (BMI)										
<25 kg/m^2^ (not overweight or obesity)	2,018	0.4	1,241	0.7	728	0.3	23	0.5	26	0.2
25-29 kg/m^2^ (overweight)	106,788	23.4	37,551	22.5	65,770	24.5	1,032	21.6	2,435	15.7
≥30 kg/m^2^ (obesity)	347,148	76.1	127,899	76.7	202,506	75.3	3,731	78.0	13,012	84.1
Eligibility category^e^										
Blood test										
Yes	211,148	46.3	103,257	62.0	92,022	34.2	2,442	51.0	13,427	86.8
No	244,806	53.7	63,434	38.0	176,982	65.8	2,344	49.0	2,046	13.2
Gestational diabetes mellitus (GDM)										
Yes	37,676	8.3	7,556	4.5	29,037	10.8	245	5.1	838	5.4
No	418,278	91.7	159,135	95.5	239,967	89.2	4,541	94.9	14,635	94.6
Risk test										
Yes	352,026	77.2	99,847	59.9	246,605	91.7	3,291	68.8	2,283	14.8
No	103,928	22.8	66,844	40.1	22,399	8.3	1,495	31.2	13,190	85.2
Entered program with blood test or history of GDM	235,038	51.5	107,157	64.3	111,333	41.4	2,628	54.9	13,920	90.0
Entered program with risk test only	220,916	48.5	59,534	35.7	157,671	58.6	2,158	45.1	1,553	10.0

^a^A participant's eligibility was based on the results of a blood-based test (A1c, fasting blood glucose (FBG), or oral glucose tolerance test (OGTT)), score on the Centers for Disease Control and Prevention's (CDC) or the American Diabetes Association's (ADA) prediabetes risk test, or by a history of gestational diabetes mellitus (GDM). ^b^Participants must have been enrolled between January 2012 and December 2019 in a program and attended at least one session. ^c^Sex was not reported for 588 (0.1%) participants. ^d^Ethnicity was not reported for 37,581 (8.2%) participants. ^e^Categories are not mutually exclusive.

## Data Availability

The data were collected under CDC's DPRP (OMB No. 0920-0909), for the primary purpose of evaluating the performance of organizations offering the National DPP lifestyle change program. Data are shared in aggregate form to inform technical assistance and enhance overall program outcomes.
